# Genomic diversification and tailocin-mediated competition in animal-associated *Xenorhabdus* bacteria

**DOI:** 10.1093/ismeco/ycag050

**Published:** 2026-03-09

**Authors:** Sarah J Kauffman, Ryan M Awori, Emmanuel C Allwell, Aaron Taylor, Farrah Bashey, Heidi Goodrich-Blair

**Affiliations:** The University of Tennessee Knoxville, Department of Microbiology, Knoxville, TN 37996, United States; The University of Tennessee Knoxville, Department of Microbiology, Knoxville, TN 37996, United States; Elakistos Biosciences, Nairobi, 00100, Kenya; The University of Tennessee Knoxville, Department of Microbiology, Knoxville, TN 37996, United States; The University of Tennessee Knoxville, Department of Microbiology, Knoxville, TN 37996, United States; Northeastern University, Department of Chemical Engineering, Boston, MA 02115, United States; Indiana University, Department of Biology, Bloomington, IN 47405, United States; The University of Tennessee Knoxville, Department of Microbiology, Knoxville, TN 37996, United States

**Keywords:** genome diversification, mobilome, intraspecific competition, tailocins, receptor binding domains, *Xenorhabdus*, glycosyltransferase, xenorhabdicins, bacteriophages, phage tail-like bacteriocins

## Abstract

Biotic interactions, including competition among bacteria mediated by phage-derived weapons, can profoundly shape microbial genomes. We found that compared to genomes across domain *Bacteria*, genomes from the animal-associated *Xenorhabdus* genus contain among the highest proportions of phage-related genes, and variation among strains in their total number of protein-coding genes was largely predicted by variation in total number of non-cargo phage genes per genome. A universal yet highly variable *Xenorhabdus* phage-related locus encoded xenorhabdicin tailocins, a key weapon in bacterial competition. The xenorhabdicin tailocin locus ranged in length from 12 to 41 kilobases and varied markedly within species. Concomitant with this variation, xenorhabdicins produced by six strains of *X. nematophila* differed in killing profiles towards each other. Mutants from two *X. nematophila* strains whose tailocin tail fibre genes were deleted lost their killing ability, while complementation experiments restored or shifted killing profiles, demonstrating that the tailocin locus is responsible for intraspecific killing and the tail fibre gene its specificity. We demonstrated the ecological importance of xenorhabdicin diversity by significantly associating broad differences in intraspecific killing profiles of mitomycin-induced cell lysates from 37 regionally sympatric *X. bovienii* strains with genes within the tailocin locus. The diversity of these genes and their rearrangements within the locus challenges our understanding of tailocin mechanics. We propose that frequent coinfection of insect hosts by multiple *Xenorhabdus* strains promotes strong selection for within-host competitive dominance as well as opportunities for genomic rearrangements, making this genus a valuable resource for examining bacterial evolution in an ecological context.

## Graphical Abstract

**Graphical Abstract:**
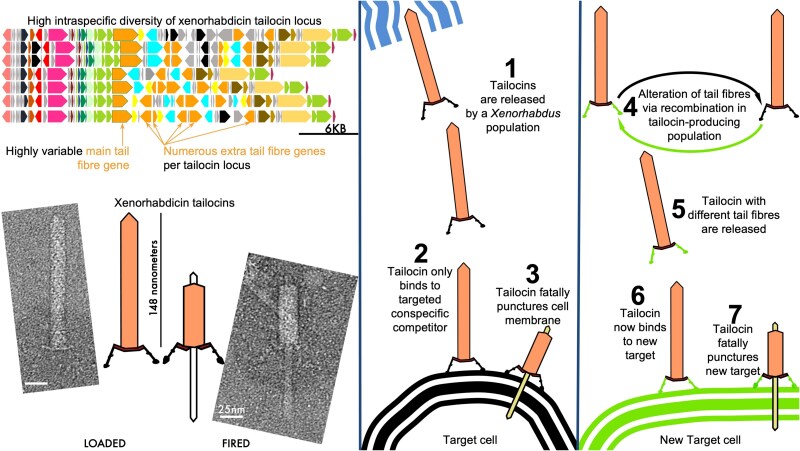

## Introduction

Diversification and adaptation of microbial lineages is facilitated by their flexible genomes, with key variation arising through both horizontal gene transfer and rearrangements from transpositions and site-specific recombination [1, 2]. Genome diversification hot spots are often those mediating interactions with eukaryotic hosts and bacteriophages [3–5], whilst antagonistic interactions among microbes themselves are an equally important driver of diversification [6, 7]. Microbes encode myriad machineries, ranging from small diffusing molecules to elaborate phage-derived nanoparticles, that can be deployed during competition with each other [8]. A single bacterium may have an arsenal of attack tactics which can be differentially deployed depending on ecological context [9]. Countermeasures against such competitive weapons and concomitant costs have been implicated in maintaining diversity within microbial populations and their genomes [10–14]. Discerning the reciprocal relationship between naturally occurring microbial competition and genome evolution affords an avenue to develop robust antimicrobials as well as methods to manipulate microbial communities [15, 16].

The bacterial competitive arsenal includes extracellular rigid contractile (R-type) phage tail nanoparticles, hereafter referred to as R-type tailocins [17–20], that terminally puncture membranes of target bacteria. Certain genera [21] biosynthesize tailocins in response to either unknown natural cues [22–24] or artificially induced DNA damage [25–27]. Tailocin locus-encoded enzymes then explosively lyse the producing cell’s envelope [24], resulting in the release of as many as 600 particles [28]. Released R-type tailocin particles consist of an ensheathed tail tube i.e. attached at its base to the centre of a hexagonal structure called the baseplate [29]. Beneath the baseplate, on the underbelly of the tail tube, is the tail spike, a conical-shaped protein that pierces the target cell membrane. Each of the baseplate’s six vertices is attached to a trimeric protein called a tail fibre. Among R-type tailocins, tail fibre homologs display variability in length and sequence even among those from the same genus or species [13, 19, 30–32]. Additionally, *Pseudomonas chlororaphis* [33] and *Pectrobacterium carotovora* [25] clones release tailocin particles with one of several different types of tail fibres, although encoded by the same tailocin locus. Binding of tailocins to specified target cells is ensured by the docking of the tail fibre C-terminal receptor binding domain (RBD) onto its specific surface receptor. In *Burkholderia cenocepacia* [28] and *Pseudomonas* spp. [12, 30, 34–36], these receptors are glycans in either the core or O-antigen regions of the lipopolysaccharide (LPS) layer. Receptor binding triggers sheath contraction [29], forcing the tail spike into the target cell to a depth sufficient to perforate the plasma membrane and permanently perturb its polarity [37]. Since self-resistance is suggested to be due to a lack of cognate glycan receptors on surfaces of the tailocin-producing population [30], LPS tailoring glycosyltransferases are pivotal players in conferring tailocin resistance [12].

Tailocins are important mediators of competition among *Pseudomonads* in natural leaf surface, seed, and rhizosphere ecosystems [13, 33, 38, 39]. Within plant-associated *Pseudomonas* communities, tailocin loci are recombination hotspots that are co-diversifying with the loci encoding LPS glycan receptors, resulting in discrete tailocin activity and sensitivity groups that are independent of taxonomic lineage [30, 38, 40]. An analysis of historic and extant populations of pathogenic *P. viridiflava* associated with *Arabidopsis* plants, spanning 200 years, revealed conserved maintenance of tailocin loci among strains that indicates the importance of tailocins for survival in this open ecosystem. A stable repertoire of variants has persisted over the centuries, suggesting that within the plant leaf surface environment, *P. viridiflava* has a limited range of genetic flexibility for evolving tailocin resistance [13]. Tailocins also appear to be mediating competition among animal pathogens, such as clinical isolates of *P. aeruginosa* in the lungs of cystic fibrosis patients [41], and pandemic strains of *Escherichia coli* [42].

Tailocins also have been established as mediators of competition for *Xenorhabdus* spp., beneficial bacterial symbionts of entomopathogenic *Steinernema* nematodes [17, 20]. In this obligatory association the nematode carries its specific *Xenorhabdus* bacterial symbiont between insect prey. *Xenorhabdus* help kill the insect, then consume it to increase their own biomass, while also protecting the cadaver from competitors and predators [17–19, 43–45]. The *Xenorhabdus* biomass is then consumed by the bacterivorous nematodes [46, 47]. The importance of *Xenorhabdus* R-type tailocins, known as xenorhabdicins, in this lifecycle was established for *X. nematophila* ATCC19061, which naturally produces and releases tailocins within the insect cadaver, killing co-infecting competitor bacteria and enabling the competitive dominance of the native nematode host of *X. nematophila* [17]. Similar tailocin function in competitive dominance has been suggested for *X. nematophila* F1 [48] and *X. bovienii* [19, 45, 49].

Xenorhabdicin tailocins are encoded by the *xnp1/xbp1* locus [17, 49] that varies in length across genomes, due to a highly variable region enriched with tail fibre genes that encode RBDs, the likely determinants of the killing spectrum of a xenorhabdicin nanoparticle [19]. This variability may reflect the intense intraspecific competition arising from *Xenorhabdus’* complex symbiotic lifestyle, which consists of repeated cycles of virulence, rapid growth, and persistence, each of which influence genome evolution [50]. In contrast to the *P. viridiflava-Arabidopsis* association, in which a community of *P. viridiflava* strains coexist on the surfaces of plant leaves, *Xenorhabdus* bacteria experience an obligatory and recurring genetic bottleneck and nutrient limitation during their transmission by individual nematodes between insect prey [51–53]. Most *Steinernema* require infection by both male and female nematodes. Since each individual nematode carries its own largely clonal *Xenorhabdus* population [52, 53], every newly infected insect comes with the potential for competition between *Xenorhabdus* strains of the same species, the outcome of which can have impacts on the fitness of the nematode-bacterium complex [54]. In studies including intra- and inter-species competitions, *Xenorhabdus* strains which grow faster than or inhibit the growth of other strains in vitro are also competitively dominant in vivo [55–57] which benefits their nematode host [58]. Finally, the two obligate behaviours of *Xenorhabdus,* nematode mutualism and insect virulence, can impose opposing selective pressures for *Xenorhabdus* genome evolution [59]. Overall, the features of this obligate beneficial symbiosis are likely to have distinctive impacts on competition as a selective force in bacterial genome evolution.

In the current study, we examine genomic diversity within the framework of this complex animal-microbe symbiosis by describing mobilome and tailocin composition across the genus *Xenorhabdus*. Using both molecular genetics and genome-wide association approaches we establish the functional importance of the tailocin locus in mediating within-species growth inhibition. Our work demonstrates that *Xenorhabdus* possesses a high level of phage-derived genomic variation and links intraspecific variation within the ubiquitously present, phage-derived tailocin region to within-species antagonisms. Thus, our work strengthens the view that competitive interactions among closely related microbes is an important driver of their genomic variation and evolutionary diversification.

## Materials and methods

### Comparative genome analyses

We constructed a pangenome of the genus from 97 publicly available genomes (Supplementary File 1) using Anvi’o 8 [60] as previously described [61] with the following crucial parameters: Markov clustering (MCL) inflation of two; use of DIAMOND [62] to calculate amino acid (aa) sequence similarities and exclusion of partial gene calls. To compute average nucleotide identities (ANIs) we used fastANI [63] and created a dendrogram from pairwise ANI values by hierarchical clustering, using the Euclidean distance metric and Ward Linkage method. Identified CDS were orthologously annotated against the NCBI COG 2019 database [64], which was also used to define the mobilome (Supplementary File 1). Using a tabulated pangenome output (Supplementary File 1) we calculated the number of genes in mobilomes, groups of orthologous genes/protein families that we termed as gene clusters (GCs), and their distribution in the pangenome's core, accessory and strain-specific regions.

For the 97 genomes, we determined any significant associations between mobilome and proteome size by calculating the Pearson's correlation coefficient between the two. Further, we examined the correlation between the size of each of three types of mobilome and the total number of protein-coding genes per genome. Prophage/phage-like loci were identified with geNomad [65]. Xenorhabdicin-encoding loci were identified by querying NCBI-annotated genomes for loci flanked by *araC* and *ogrK.* Loci were reannotated using Bakta [66], Pharokka [67] and RASTk [68], visualized in Geneious 8.6.,1, and used to create gene diagrams as well as assign predicted functions that were supported by more than one annotation pipeline. For phylogenomic analyses, digital DNA–DNA hybridization distance trees were reconstructed as previously described [61]. Tree comparisons were made using normalized Robinson-Fould distances in ETE3 [69]. Maximum likelihood tree based on main tail fibre protein sequences of 97 strains was made on IQTREE server [70] with a Probability Matrix from Blocks substitution model and 772 parsimony informative sites.

Genome wide association analyses were conducted to identify GCs associated with the killing spectra of *X. bovienii* using a binomial general linear model, Rao test and calculation of *q*-values from *P*-values as explicated in a previously described workflow [71].

### Tailocin induction and visualization in *X. nematophila* strains

Six sequenced strains of *X. nematophila* were available for empirical characterization of tailocin morphology and killing activity. To induce tailocin production in these six *X. nematophila* strains, 100 ml LB was inoculated with overnight cultures (5%) at an OD_600_ of 0.05. Cultures were then grown to an OD_600_ of 0.5–0.6, at which point tailocin production and release was induced using mitomycin C at a final concentration of 5 μg/ml followed by incubation at 30°C with shaking for 20 h. RNase A and DNase I were added at final concentrations of 1 μg/ml, and cultures were incubated for 30 min at 37°C with shaking, before centrifugation at 3500 × ***g*** for 20 min to pellet cellular debris. Supernatants were filtered through 0.45 μm pores and incubated at 4°C for 4 h at a final concentration of 1 M NaCl and 10% polyethylene glycol (PEG) 8000. Following incubation, filtrates were centrifuged at 13 750 × ***g*** for 15 min. Supernatants were poured off and trace amounts were aspirated with a pipette. Precipitates were resuspended in 3 ml LB and centrifuged again at 6000 × ***g*** for 5 min. The final solution was filtered through 0.2 μm pores and its protein concentration determined using Pierce 660 nm Protein Assay Reagent. Tailocin preparations were cryoprotected with glycerol (4%(v/v)) before storage at 4°C.

For transmission electron microscopy (TEM) preparation, 200 μl of tailocin preparation was normalized to an A_280_ reading of 0.25, then ultracentrifuged at 287 500 × ***g*** for 15 min. The supernatant was removed, and pellets were resuspended in 50 mM Tris–HCl, pH 8.7. A TEM negative staining protocol was followed. Briefly, a carbon coated copper grid (CFT200-Cu-50, Electron Microscopy Science) was placed over a 20 μl drop of tailocin sample, rinsed in distilled water and stained with Uranyless (Electron Microscopy Sciences). Imaging was performed using a JEOL 1400Flash TEM (JEOL USA, Peabody, Massachusetts), and all images captured at 120 kV.

### Construction of *xnpH1* and *xnpS1* deletion mutants

To test whether x*np1* locus genes are necessary for tailocin production by *X. nematophila* ATCC19061 and Anatoliense strains, allelic exchange was used to make unmarked deletions of *xnpH1* (main tail fibre) and *xnpS1* (sheath) tailocin genes. The procedure is given in the Supplementary Methods and Tables.

### *xnpH1* complementation

To complement *xnpH1* of ATCC19061 in *X. nematophila* ATCC1901-Δ*xnpH1* and Anatoliense-Δ*xnpH1* mutants, pEVS107 donor plasmid was modified to deliver *placUV5*-*xnpH1* (*xnpH1* under the constitutive *placUV5* promoter) into the chromosomal *att*Tn7 site of the two mutants. The procedure is given in the Supplementary Methods and Tables.

### Bacterial growth conditions

All *Xenorhabdus* strains were cultured in Luria Bertani (LB) media consisting of tryptone (10 g/L), yeast extract (5 g/L), and NaCl (10 g/L), adjusted to pH 7.0 and, when needed, solidified by supplementation with agar (20 g/L). Unless otherwise noted, *X. nematophila* were grown at 30°C and *X. bovienii* were grown at 28°C. *E. coli* strains with the pKNGkan vector were grown at 37°C in LB broth supplemented with kanamycin (50 μg/ml). The *E. coli* S-17 λ pir diaminopimelic acid (DAP)-dependent strain was grown at 37°C in LB broth supplemented with DAP and kanamycin. *E. coli* transformants were cultured on LB agar plates containing DAP and kanamycin. For the selection of recombinant strains, LB agar (15 g/L) plates supplemented with sucrose (50 g/L) were used. Exconjugants were selected on LB agar plates supplemented with kanamycin (50 μg/ml). All the bacterial strains and plasmids used in deletion experiments in *X. nematophila* are listed in Table S1 and primers are listed in Table S2, both in the Supplementary Methods and Tables.

### Growth inhibition assays using tailocin preparations of mitomycin-induced cell lysates from *X. nematophila*

To assess intraspecific tailocin activity, tailocin preparations from MILs of each strain were tested for growth inhibition against all other strains in liquid growth assays. Overnight cultures of recipient strains were diluted to an OD_600_ of 0.05 in 5 ml LB then grown to an OD_600_ of 0.5–0.6 and diluted 1:750. In a 96 well plate, 100 μl of diluted culture was combined with 50 μl of tailocin preparation from the actor strain. For negative controls, 50 μl of LB with 4% (v/v) glycerol was used instead of tailocin preparations. All inhibition assays were conducted in technical triplicates. Percentage growth inhibition was calculated as (1-(OD treatment/OD control)) *100 (Supplementary File 2). The ideal time point selected for the OD reading was when the control treated culture first plateaued in stationary phase.

### Growth inhibition assays of mitomycin-induced cell lysates from *X. bovienii*

The killing and susceptibility spectra of 42 sympatric and sequenced *X. bovienii* strains [72] were determined by soft-agar inhibition assays of recipient strains exposed to MILs from actor strains (Supplementary File 1). Briefly, 5 ml LB broth (Difco) in 20 ml culture tubes was inoculated with individual colonies of each strain picked from freezer stocks. Cultures were grown overnight and then used to inoculate LB broth, wherein bacteria were cultured up to an OD_600_ of 0.5, at which point mitomycin C (Sigma-Aldrich) was added at a final concentration of 0.5 μg/ml to induce both tailocin production and release through cell lysis [17, 18, 20]. After overnight incubation, mitomycin-induced cell lysates (MILs) were obtained by centrifuging cultures at 4472 × ***g*** for 15 min then filtering the supernatant through 0.45 μm pore-sized membranes (Acrodisc). MILs were stored at 4°C until use.

The inhibitory activity of each MIL was tested by spotting 10 μl onto soft nutrient agar (5 g/L) sowed with 2% (v/v) of stationary-phase liquid culture of a recipient colony. Plates were incubated for 48 h, when inhibition could be visualized as a clear zone on the recipient lawn. Assays were scored on a 0–3 scale, with 0 indicating no sign of inhibition, and averaged between two observers. Supernatants from blank LB cultures treated with mitomycin C and self-tests, whereby both the MIL and the recipient colony were of the same strain, were used as negative controls. Two independent inductions were performed for each strain, and each actor was tested against at least 16 recipients. Replicate assays for the same actor-recipient pair were averaged. Using SAS v. 9.4, the calculation of phenotypic distances between killing profiles for each strain was performed using the Euclidean method, while hierarchical clustering was performed using Ward’s Minimum Variance Method. In total, 37 strains were characterized for their killing, and 39 strains for their susceptibility based on 3249 inhibition tests.

## Results

### Six percent of a *Xenorhabdus* genome is mobilome

**Figure 1:**
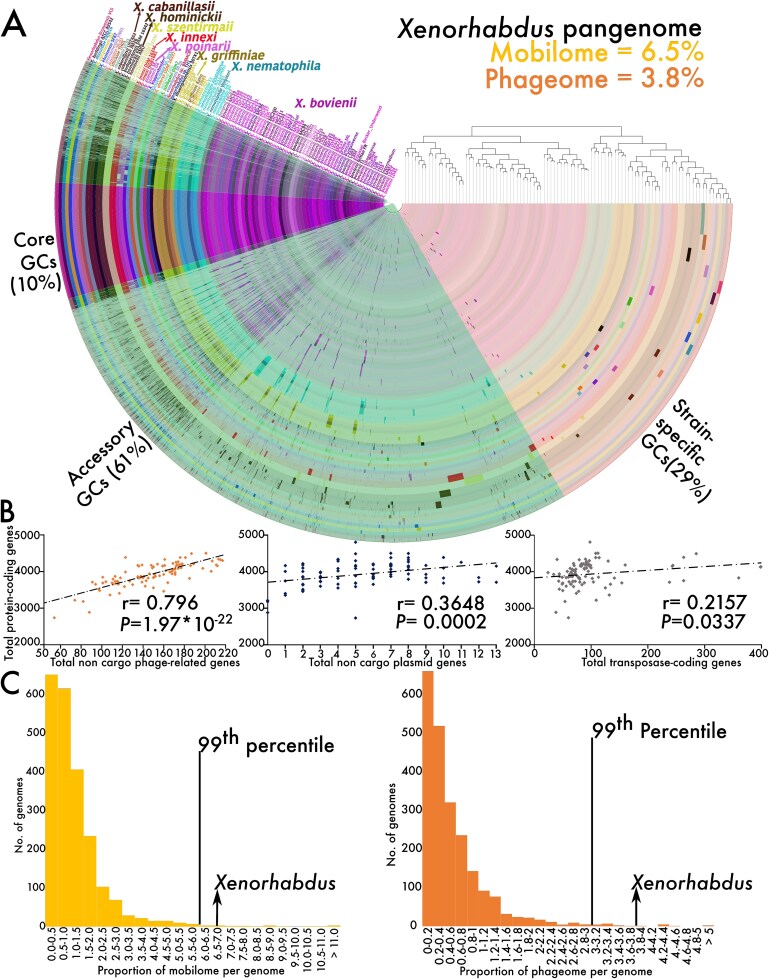
*Xenorhabdus* bacteria have comparatively large mobilomes, which are associated with genome diversification. (A) Graphical representation of a pangenome of 97 strains of 27 species from the genus *Xenorhabdus*, and its mobilome proportion. Each of the 97 concentric arcs represents a genome. Each radius represents a group of orthologous genes (gene cluster). Dark shades along a radius indicate the presence of a gene cluster (GC) in the genome. The eight species with more than one strain in the pangenome are shown in large font. The pangenome was composed of 381 400 protein coding genes grouped into 15 644 GCs. There were 1520 core, 9562 accessory and 4562 strain-specific GCs, where we defined core, accessory, and strain-specific GCs as those constituted of orthologs from *n* = 97, *n* = 2–96, and *n* = 1 genomes, respectively. The dendrogram at the top is based on hierarchical clustering based on presence and absence of GCs. (B) Scatterplots depict correlations between components of the mobilome and the proteome (total number of protein-coding genes) sizes in 97 *Xenorhabdus* genomes. Each point in the graphs represents a genome. The greatest proportion of the variation in total protein-coding genes in genomes was accounted for by the number of phage-related genes per genome. (C) *Xenorhabdus* genomes have among the highest mobilome proportions among sequenced prokaryotic genomes. The histograms depict the mobilome and non-cargo phage genes proportions in 2196 circularized bacterial and archaeal genomes.

To characterize genome diversification among *Xenorhabdus*, we used the genomes of 97 strains from 27 species to create a pangenome that contained 381 400 protein-coding genes that formed 15 644 orthologous groups of genes/protein families (GCs) (Fig. 1A). Core genome GCs were only 10% (Fig. 1A) indicative of an open pangenome [43], while strain-specific GCs were 29% indicative of high pangenome fluidity characteristic of motile extracellular facultative symbionts [73] like *Xenorhabdus*.

The average genome length, guanine-cytosine (G + C) content, and total number of protein-coding genes for a *Xenorhabdus* genome were 4.54 megabases (Mb), 44.33%, and 3944, respectively. Strikingly, the ranges for the total number of protein-coding genes and genome length were 2734–4803 and 3.18–5.35 Mb, respectively, demonstrating considerable genome diversification within the genus. Variation across genomes in the total number of protein coding genes was significantly correlated (*r* = 0.5579, *P* = 4.17*10^−09^) with the size of the mobilome, which comprised 6.54% of the pangenome. Notably, variation in total number of non-cargo phage-related genes explained 63% of the variation in proteome size (Fig. 1B).

With six percent of their genomes being mobilome, *Xenorhabdus* bacteria were in the top 1% of strains whose genomes contain the largest mobilome proportions in general and specifically non-cargo phage genes, when compared to those of 2196 annotated prokaryotic genomes available in the NCBI COG 2024 database (Fig. 1C). Examining the phage gene proportions across these genomes, there was no clear similarity in taxon or habitat among species in the top 1%.

### The xenorhabdicin tailocin locus exemplifies phage-associated genome diversification

To examine the diversity of phage-related genes in *Xenorhabdus*, we chose the 10, least-fragmented genome assemblies that were most phylogenetically diverse in our dataset and identified their prophage/phage-like regions (Fig. S1). On average, each genome had eight prophages, which is between previous estimates of six [74] and nine [75]. These regions accounted for 6.5% of the genome’s base pairs and contained 9.8% of its total protein-coding genes. Sixteen distinct clades of prophage/phage-like regions were found across the genomes, with some clades showing diversification both across and within *Xenorhabdus* species (Fig. S1). The clade with the least diversity had only one member, the *xnp1/xbp1* locus [17, 49], from each of the 10 genomes (Fig. S1). We subsequently found it in all 97 *Xenorhabdus* genomes, indicating that it encodes a core *Xenorhabdus* function and prompting us to focus on it further.

**Figure 2:**
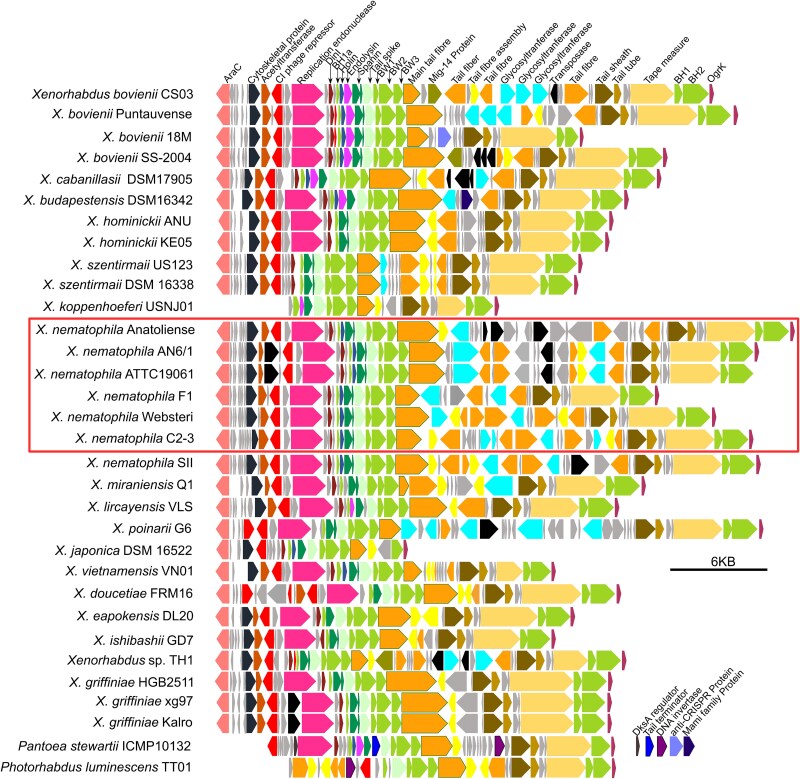
The xenorhabdicin-encoding locus contains both conserved and variable regions. An alignment of this locus in 30 strains of *Xenorhabdus* compared to previously characterized tailocin-encoding loci in *Photorhabdus* and *Pantoea*. Apart from those of *X. nematophila* FI, Websteri, Anatoliense C2–3, and SII, all shown genome loci were found on one contig and were not split by putative transposition events. The shown loci for Websteri and C2–3, Anatoliense, were reconstructed by splicing more than two contigs together, and therefore are likely to change with better assemblies. The names of the main proteins which are predicted to be encoded by the genes are in the topmost row. Grey indicates that the gene encodes a protein whose predicted function remains unknown. The six *X. nematophila* strains in the red box are those whose tailocin activity was further investigated.

Xenorhabdicin biosynthesis is encoded by the *xnp1/xbp1* locus [17, 49], which ranged in length from 12.2 kilobases (Kb) in *X. japonica* to 41.3 Kb in *X. bovienii* LD8 (Fig. 2, Fig. S2). The length variation was primarily due to differences in gene content in a highly variable region that lies between the tail sheath gene and the gene fragment that encodes the N-terminal of the main tail fibre (Fig. 2). Conserved genes that encode proteins with high pairwise identity across the genus flanked the variable region. These include those encoding a spanin; transcriptional regulators AraC, OgrK, CI phage repressor; the SOS response regulator DinI; baseplate hub proteins BH1a, BH1, baseplate wedge proteins BW1, BW2 and BW3 [76]; and the tail spike, sheath and tube (Fig. 2). Although two genera, *Photorhabdus* and *Pantoea,* that are phylogenetically close to *Xenorhabdus* also have loci that encode rigid contractile tailocins, photorhabdicin and pantailocin [32, 77], respectively, xenorhabdicin differed from these. The pairwise identity of structural proteins such as BW2 and tail sheath was much higher within *Xenorhabdus* (96% and 93%, respectively) than with their cognates in photorhabdicin (74%, 88%) or pantailocin (69%, 73%). Moreover, although *Xenorhabdus* and *Photorhabdus* share an ecological niche, the collinearity of genes in the xenorhabdicin locus was more like that of the pantailocin locus (Fig. 2).

**Figure 3:**
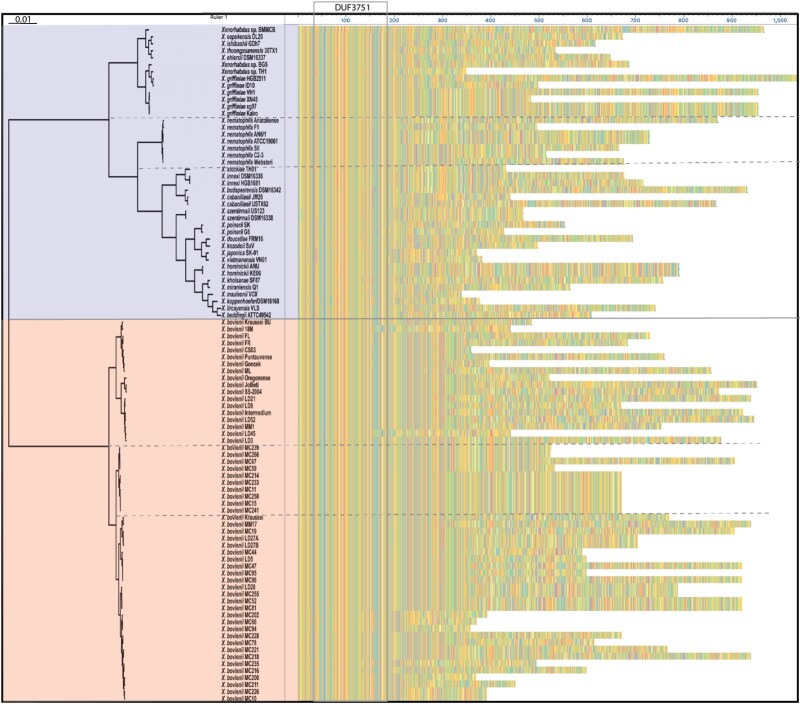
Amino acid sequences of xenorhabdicin main tail fibres (XnpH1) from 97 strains of 27 species from the genus *Xenorhabdus* share a conserved *N*-terminus but have variable middle and C-terminal coding regions. On the left is a dendrogram depicting the overall genome relatedness among these 97 strains based on average nucleotide identities (ANI) values. The 97 strains fall into two distinct clades, highlighted with blue and pink shading, respectively. The dashed lines separate additional clades within the genus. Shown on the right are the XnpH1 sequences aligned using DNAStar Megalign software to show regions of amino acid similarity and diversity. The colour scheme represents amino acid side chain chemistry: aromatic = yellow; acidic = red; basic = blue; non-polar = brown; polar = green. The *N*-terminal coding region is highly conserved across all 97 XnpH1 and comprises the ~160 amino acid DUF3751, which is predicted to function in attaching the tail fibre to the baseplate of the tailocin. The C-terminal regions are predicted to contain receptor binding domains (RBDs) that mediate attachment to target cell surfaces. A high level of variability is observed in these RBDs, even among strains of the same species, that in turn likely reflects variability in specificity for target cell surfaces.

Although present in all 97 strains, the main tail fibre (XnpH1) varied dramatically (Fig. 3) and did not correspond to genome relatedness (Fig. S3). XnpH1 has a highly conserved N-terminal DUF3751 sequence, which is predicted to attach the tail fibre to the baseplate, and variable C-termini that are predicted to be RBD that mediate binding to target cell surfaces [19]. In the N-terminal portion, all 97 proteins were nearly identical up to 150 aa, and with just a few exceptions up to 213 aa (Fig. 3). A central region of identity was conserved among some subsets of main tail fibre proteins, with different subgroups sharing different central domains (e.g. positions 213–350 are similar among the majority of *X. bovienii* isolates). The divergence of the 97 main tail fibre C-termini, even among strains of the same species (Fig. 3), further supports their predicted function as xenorhabdicin RBDs.

### *X. nematophila* tailocins differ in intraspecies killing spectrum

**Figure 4:**
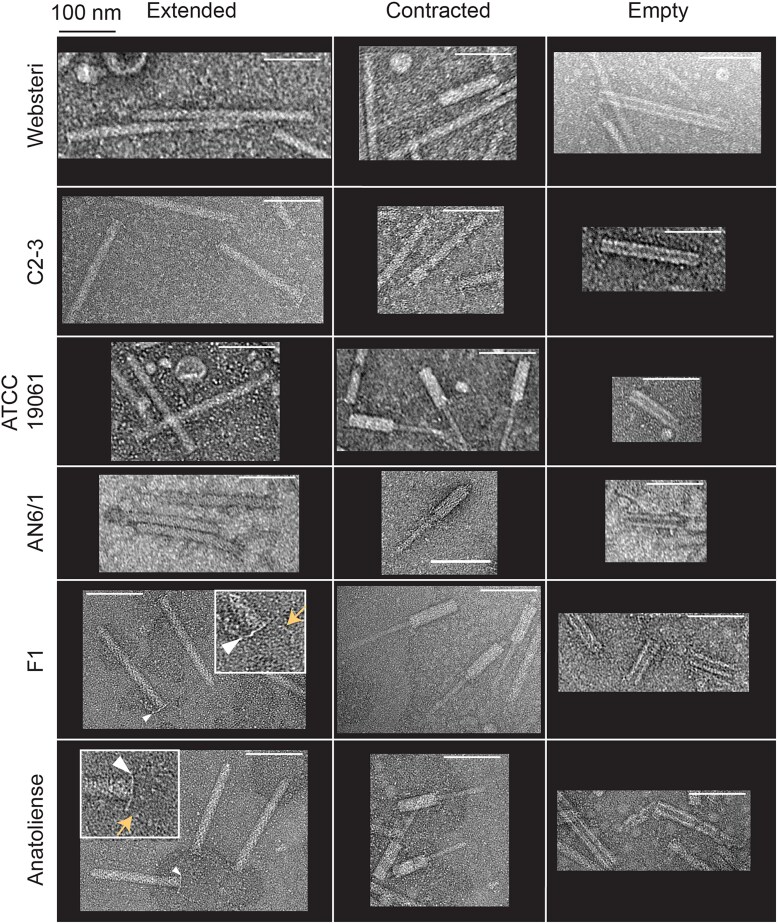
Structures of tailocins are produced by six *Xenorhabdus nematophila* strains: Websteri, C2–3, ATCC19061, AN6/1, F1, and Anatoliense. Tailocin preparations were visualized using transmission electron microscopy (TEM), with extended (pre-firing), contracted (post-firing), and empty sheath structures apparent. Representative images are shown. Scale bars represent 100 nm, indicating xenorhabdicins may be larger than tailocins seen in other genera, reported as 98–184 nm in length [11, 35]. In some extended tailocin structures, the baseplate (white arrowhead) and tail fibres (orange line-arrow) were visible. For Anatoliense tailocin i.e. highlighted, the length of the tail fibre and diameter of the baseplate were ca. 24 nm and 32 nm, respectively.

**Figure 5:**
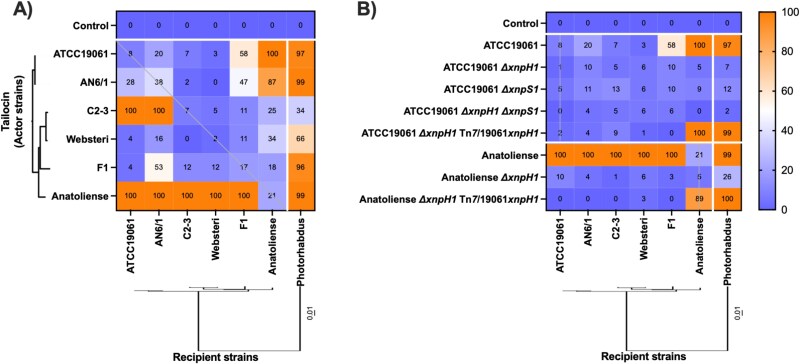
*Xenorhabdus nematophila* xenorhabdicin tailocins differentially inhibit *X. nematophila* recipient strains. Tailocins from each actor strain (Y axis) were tested for inhibition of each recipient strain in a LB liquid killing assay. Tailocin preparations were obtained from MILs of six wild type *X. nematophila* strains (A) or from *X. nematophila* ATCC19061 or Anatoliense mutants (B) in which *xnpH1, xnpS1*, or both had been deleted, or to which ATCC19061 *xnpH1* had been inserted at the *att*Tn*7* site (Tn*7/*19061*xnpH1*). Wild type ATCC19061 and Anatoliense actor strain data are identical in both panels. Tailocin activity was measured against each of the six recipient *X. nematophila* strains and *Photorhabdus luminescens* TT01 (X axis), the phylogenomic distance tree shown for the strains is based on digital DNA–DNA hybridization (dDDH). Percentage growth inhibition was measured compared to the LB only control with blue indicating no difference in OD when exposed to LB or tailocin (0 inhibition) and orange corresponding to 100% inhibition of growth of recipient strain in the presence of the tailocin. The light grey lines indicate assays of actor and recipient from the same strain background.

Having observed diverse gene content in the *xnp1* regions of the seven sequenced *X. nematophila* strains, we tested all available strains to see whether they differed in their tailocins and concomitant killing spectra. All six strains produced visible tailocin structures (Fig. 4). For F1 and Anatoliense, the baseplate and tail fibres could be seen clearly. No other phage-like elements were detected (Fig. 4). Each actor strain tailocin was assayed for its level of inhibition against each of the other six *X. nematophila* strains (Fig. 5A). Anatoliense tailocins exhibited the highest level and broadest spectrum of killing activity, while Websteri and F1 tailocins had little to no inhibitory activity against the other *X. nematophila* strains. AN6/1 and ATCC19061 shared similarity in their tailocin loci (Fig. 2), *xnpH1* sequences (Fig. 3) and killing profiles (Fig. 5A). Additionally, both were highly susceptible to the tailocins produced by C2–3, which inhibited no other strains.

The dependence of killing activity on an intact *xnp1* locus was tested by creating *xnpH1* (main tail fibre) deletion mutants in ATCC19061 and Anatoliense. In addition, we created *xnpS1* (tail sheath) and *xnpH1/xnpS1* double mutants in ATCC19061. Consistent with previous findings [17], mutants lacking *xnpH1* or *xnpS1* or both no longer produced tailocin particles (Fig. S4) and were unable to kill other strains (Fig. 5B), indicating that the *xnp1* locus encodes the observed tailocin killing activity. Furthermore, complementation of the tail fibre mutant of ATCC19061 with its own *xnpH1* restored its killing profile (Fig. 5B). Strikingly, when Anatoliense *xnpH1* mutant was complemented with the tail fibre gene of ATCC19061, its killing spectra mirrored that of ATCC19061 rather than Anatoliense (Fig. 5B), demonstrating that the main tail fibre gene is necessary and sufficient for killing specificity.

### Sympatric isolates of *X. bovienii* show distinct killing profiles that are underpinned by tailocin loci

**Figure 6:**
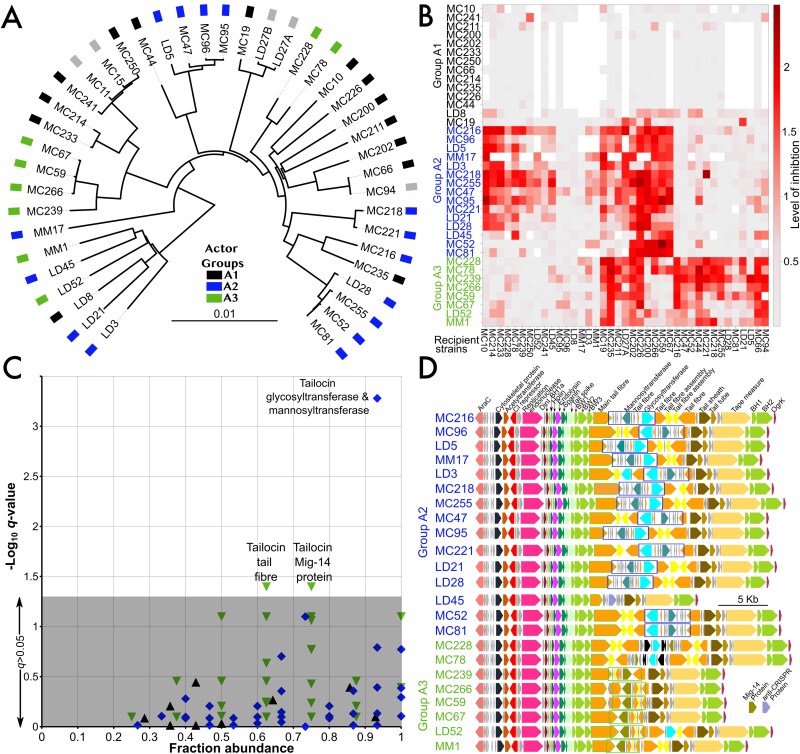
The diversity in killing spectra of mitomycin-induced lysates (MILs) from sympatric *Xenorhabdus bovienii* is underpinned by tailocin loci. (A) Phylogenomic relationships of 42 strains of *X. bovienii* that were isolated from soil biota from three forest sites (MC, MM, LD) from a 240 km^2^ area in Indiana, USA. This distance tree is based on digital DNA–DNA hybridization pairwise distances. For each strain, actor phenotypes are mapped as colour codes, a grey box indicates that the strain was not phenotyped. (B) Killing spectrum of each *X. bovienii* mitomycin-induced lysate (MIL). The leftmost column contains names of *X. bovienii* strains from which MILs were produced, clustered based on their actor phenotype. The bottom row contains the names of *Xenorhabdus* strains against which inhibition assays were conducted. Along a row, the colour of the cell indicates the level to which the MIL produced by strain in the leftmost column, inhibited the strain in the bottom row, with deep red being strong and grey being no inhibition. (C) Significance of genome wide associations between each actor group (A1 = black, A2 = blue, A3 = bright green) and genes against the relative frequency of each gene in an actor group. The proteins predicted to be encoded by the genes that were significantly associated are given. (D) Alignment of xenorhabdicin tailocin-encoding loci of strains from actor group A2 and A3 highlighting regions of percentage pairwise identities >98% that were significantly associated with killing spectra. For the group A2, a 4.6-kb locus was uniquely found among 14/15 strains while for A3 a 3-kb locus was uniquely found in 6/8. For both these loci, they were highly identical, often inverted and encoded tail-fibre receptor binding domains.

We next investigated the ecological role of putative xenorhabdicin tailocins in intraspecific killing. We focused on a set of 42 *X. bovienii* (Fig. 6A) because they had been previously isolated from three forest communities found in a 240 km^2^ area and had sequenced genomes [72]. We obtained their mitomycin-induced cell lysates (MILs) and quantified how much each strain was inhibited by another’s MIL (Fig. 6B). We then clustered these strains into actor groups based on whether their MILs generally killed the same recipients. This resulted in three actor groups: A1, A2, and A3 (Fig. 6B). None of the actor groups can be considered monophyletic as can be seen by their mapping on the phylogeny (Fig. 6A).

To identify GCs that are significantly associated with the killing spectra of MILs, we first identified those that are uniquely found in one of the three actor groups (Fig. 6C). All but one of the genomes of strains from actor group A2 contained two GCs that were absent from the other genomes. These two GCs coded for a glycosyltransferase and a mannosyltransferase and were both found in the *xbp1* highly variable region (Fig. 6D). To increase sensitivity to viral gene calls [78], we redid the association analysis using only Pharokka-annotated *xbp1* loci and identified an additional seven GCs which had not been previously called and that were significantly associated with A2 genomes (Fig. 6D). These nine GCs that were significantly associated with A2 genomes were in the same locus (Fig. 6D).

Multiple sequence alignment of the nine GCs revealed a 4.6-Kb fragment that had a percentage pairwise identity of 98% for the 14 A2 genomes (Fig. 6D), notwithstanding its location in the highly variable *xbp1* region (pairwise identity of 25% for all 42 *X. bovienii* strains). For eight A2 genomes, this 4.6-Kb locus contained the C-terminal encoding fragment of the main tail fibre gene (Fig. 6D) demonstrating a direct and significant association (*q-value* = 0.00053) between the tail fibre RBD and killing spectra. For the remaining six A2 genomes, the orthologous tail fibre gene fragment was of an extra tail fibre gene, showing that extra tail fibre genes are significantly associated with tailocin killing spectra. For these six genomes, only when the entire 4.6-Kb fragment was inverted did it align with the eight (Fig. 6D). These inversions demonstrate the dynamic nature of this region as sister taxa whose pairwise ANI values were > 99.98% (Supplementary File 1) often differed in gene orientation (e.g. MC255 vs. MC52, MC95 vs. MC96, MC221 vs. MC218).

We additionally identified two GCs that were significantly associated with A3 killing spectra (Fig. 6C). These genes were also found in the tailocin locus, respectively encoding a tail fibre and Mig-14 protein. Following the steps described in elucidating the 4.6-Kb locus in the A2 genomes, we similarly elucidated a 3-Kb locus with a 98% pairwise identity that was uniquely found in the highly variable tailocin-encoding region of six A3 genomes (Fig. 6D). This region encoded RBDs of both extra and main tail fibres and was inverted between sister taxa (MC59 vs MC67). Together, these findings indicate that tailocins and DNA inversions are associated with competitive interactions among *X. bovienii*.

## Discussion

The association of genome evolution with functional diversity is crucial for understanding the ecological forces that shape microbial lineages. Our comparative analysis of 97 *Xenorhabdus* genomes revealed that they have among the highest proportion of phage-related genes found in bacteria (Fig. 1C). These genes underlie striking variation in proteome size across *Xenorhabdus*, with 9.8% of protein-coding genes found in phage-associated regions. The correlation of non-cargo phage genes with proteome size (Fig. 1B) indicates that phages are primary porters used by *Xenorhabdus* genomes to acquire genes. While the adaptive importance of phage-derived regions has been well established in bacterial pathogens, as many virulence factors and biological weapons used for host colonization are encoded by phage regions [79], here we show that variation in a phage-derived genomic region is associated with intraspecific competitive interactions.

All *Xenorhabdus* genomes encoded the R-type tailocin, xenorhabdicin, indicating an ancestral origin of this phage-derived region, which has evolved to range more than three-fold in size across the genus (Fig. 2). The diversity found in the xenorhabdicin-encoding locus, even within a single species, suggests it may be under selection. Indeed, xenorhabdicin is an important mediator of competition in both *X. bovienii* and *X. nematophila*, capable of protecting an insect cadaver from competing strains and benefiting the nematode host of the producing strain [17, 18]. We visualized tailocins from six *X. nematophila* strains (Fig. 4) and used inhibition assays to corroborate that differences in tailocin gene content are reflected in distinct killing profiles (Fig. 5A). Moreover, through the deletion of tail fibre and sheath genes in two *X. nematophila* strains and concomitant loss of tailocin particles and killing activity, we demonstrated the functional importance of the tailocin locus and that the tail fibre is necessary and sufficient to alter the killing target range (Fig. 5B). Finally, we demonstrated the ecological relevance of the tailocin locus by showing significant genome-wide associations between genes in this locus and the killing profiles of 37 regionally sympatric *X. bovienii* (Fig. 6C). Three distinct killing phenotypes were spread across the *X. bovienii* phylogeny (Fig. 6A), suggesting active genomic rearrangements. Forest site origin (either MC or MM or LD) of the nematode host of the *X. bovienii* strains was not congruent with tailocin killing profile, and strains collected contemporaneously from neighbouring soil samples (e.g. MC95 vs 59 or MC228 vs MC218) or even the same sample (MC266 vs MC234) produced tailocins that strongly inhibit each other (Fig. 6B). This indicates that in nature, sympatric *Steinernema* nematodes frequently co-infect the same insect [80] and may utilise their bacterial symbionts’ tailocins for competitive dominance within the cadaver [18, 57, 58, 81].

Consistent with prior studies [33, 82] our complementation experiments revealed that the tail fibre is essential for xenorhabdicin tailocin killing. Using heterologous complementation, we further show that the tail fibre is sufficient to confer a specific tailocin killing profile; when the tail fibre of one *Xenorhabdus* strain (ATCC 19061) is expressed in the background of another strain (Anatoliense) the latter adopted the killing profile of the former. This provides direct experimental evidence for the concept that tail fibre variation in receptor target specificity is solely responsible for the outcome of competition among members of the same species, as has been inferred from comparative genomics correlated with killing profiles [13, 25, 30, 82]. Altered tailocin killing target profiles upon replacement of the endogenous tail fibre with phage, chimeric, or non-native tailocin tail fibre has been reported for *Pseudomonas spp.* tailocins. In these systems, the altered tail fibre target profile required the presence of both the tail fibre and a chaperone gene [40, 83]. Similarly, *P. chlororaphis* 30–84 produces two R-type tailocins with different killing target ranges against other *Pseudomonas* species that were attributed (by mutagenesis and complementation) to different tail fibre and chaperone pairs [33]. In our work, the sufficiency of the tail fibre gene alone (without an associated chaperone) in directing target specificity against other members of the same species may indicate that in this system, tail fibre variants can utilize a common chaperone, or that chaperone activity is not essential for xenorhabdicin function.

The main tail fibre *xnpH1* gene was highly variable across the genus (Fig. 3). The XnpH1 protein ranged in length from 341 to 1035 aa, with variability occurring in the C-terminal domain, indicative of diversifying selection on its RBD. Unlike the loci that encode R-type tailocins in *Pseudomonas* [13, 36, 40], *Burkholderia* [28], *Clostridium* [84], *Dickeya dadantii* [23], a xenorhabdicin-encoding locus encodes very diverse RBDs as it contained up to 13 tail fibre and tail fibre assembly genes (Fig. S2) compared to six for *Pragia fontinum* and *Leminorella richardii* which, hitherto, had the highest number of such genes per R-type tailocin locus [85]. Extra tail fibre genes did not encode the conserved N-terminal domain of the main tail fibre i.e. expected to mediate attachment to the tailocin baseplate [19]. However, their association with killing phenotype suggests these extra fibres are incorporated into the tailocin particle. For eight *X. bovienii* strains, an extra tail fibre gene was significantly associated with killing spectra (Fig. 6C and D). When inverted, the extra tail fibre gene matched the C-terminal encoding fragment of other strains in their phenogroups. This suggests that extra tail fibre genes are exchanged with the C-terminal encoding fragment of the main tail fibre gene to alter specificity. Site-specific recombination may occur upon induction of xenorhabdicin biosynthesis, and the stochastic nature of this process may explain the variation observed within the actor phenogroups (Fig. 6B). In *Pectobacterium carotovorum* [25], site-specific recombination altered the killing spectra of their tailocins, and it has been postulated to do the same for *Pantoea stewartii* [32]. Both their corresponding loci contain DNA invertase genes, unlike any of the xenorhabdicin-encoding loci. However, all 97 genomes did contain DNA invertase genes in other loci, indicating that DNA inversion within xenorhabdicin-encoding loci may be mediated by multi-site recombinases [86].

Glycosyltransferase genes were also frequently found in the hypervariable region of the xenorhabdicin-encoding locus (Fig. 2) and were significantly associated with the killing spectra of *X. bovienii* strains (Fig. 6C). Bacterial glycosyltransferases catalyse cell envelope biogenesis, and when encoded by prophages, they are used to tailor the lipopolysaccharide layer (LPS) of a bacterium to make it nonspecific to the RBDs of bacteriophages released by sister cells [87]. Similarly, significant correlations between various tailocin tail fibre genes and *rfbD* alleles were postulated to result in a *P. syringiae* population having an LPS to which its own tailocins were nonspecific [30]. Hence, we propose that the mannosyltransferase and glycosyltransferase genes associated with the killing spectra of A2 strains mediate a self-resistance mechanism by encoding modifications to the LPS making it non-specific to the RBDs of xenorhabdicins released by a sister cell. This would explain the preponderance of glycosyltransferases genes in xenorhabdicin-encoding loci.

In conclusion, *Xenorhabdus* genomes have a high and variable phage gene content that includes the xenorhabdicin tailocin locus. Although ubiquitous across the genus, the diversity within this locus is profound. In *X. nematophila,* we empirically demonstrate that this locus is necessary for intraspecific killing and that tail fibres are determinants of tailocin killing specificity. In field isolates of *X. bovienii,* we see intraspecific killing profiles significantly associated with both tail fibre and glycosyltransferase genes in regions of the tailocin locus that are inverted across genomes. We postulate that tail fibre genes frequently undergo recombination to alter tailocin killing spectra. Our findings are a basis for investigations into how this recombination occurs, what receptors tail fibres target, and the genus’ largely unexplored phageome. Altogether, this study indicates that the rich biotic niche of animal-associated *Xenorhabdus* bacteria not only facilitates recombination but also results in diversification due to competitive interactions. Unravelling the intersections of these two phenomena may afford crucial insights into how microbial symbionts respond to the complex selective environments encountered as they associate with eukaryotic hosts.

## Supplementary Material

Supp_Text_Fig_Table_ISMECOMMUN-D-25-00705_ycag050

## Data Availability

All data generated or analysed during this study are included in this published article [and its supplementary information files].
