# Supplementary material for: Genomic diversification and tailocin-mediated competition in animal-associated Xenorhabdus bacteria
Source: ISME Commun. 2026 Mar 9;6(1):ycag050. doi: 10.1093/ismeco/ycag050 (PMC13280938; doi:10.1093/ismeco/ycag050)
Supplement: Supp_Text_Fig_Table_ISMECOMMUN-D-25-00705_ycag050 [file supp_text_fig_table_ismecommun-d-25-00705_ycag050.pdf]

# Genomic Diversification and Tailocin-Mediated Competition in Animal-Associated *Xenorhabdus* Bacteria

## Supplementary Methods, Tables, and Figures

Sarah J. Kauffman[1,*], Ryan M. Awori[2,*], Emmanuel C. Allwell[1], Aaron Taylor[1,3], Farrah Bashey[4], Heidi Goodrich-Blair[1,#]

[1]The University of Tennessee Knoxville, Department of Microbiology, Knoxville, Tennessee, USA

[2] Elakistos Biosciences, Nairobi, Kenya

[3] Northeastern University, Department of Chemical Engineering, Boston, Massachusetts, USA

[4] Indiana University, Department of Biology, Bloomington, Indiana, USA

*These authors contributed equally to the work

[#]Correspondent footnote: hgblair@utk.edu

Running title: *Xenorhabdus* Diversification and Tailocins

Keywords: Genome diversification; mobilome; intraspecies competition; tailocins; receptor binding domains; *Xenorhabdus;* glycosyltransferase; xenorhabdicins, bacteriophages, phage tail-like bacteriocins.

## Supplementary Methods and Tables

## Construction of *xnpH1* and *xnpS1* deletion mutants

We generated single tail fibre mutants in both ATCC19061 (Δ*XNC1_1193)* and Anatoliense (Δ*XNA1_v220011*). Additionally, an *xnpS1* sheath mutant and a *xnpH1 xnpS1* double mutant were created in the ATCC19061 strain background. To generate deletion constructs, primers were designed to amplify ~1 kb upstream and downstream of each gene to be deleted (Supplementary Table S2) and amplified products were cloned into the vector pKNGkan.

Amplification primers for upstream and downstream fragments included 20–30 bp overlaps homologous to sequences flanking the pKNGkan vector (Supplementary Table S2) multiple cloning site, to facilitate fusion using HiFi assembly (NEBuilder® HiFi DNA Assembly). pKNGKan is a derivative of pKNG101 [1] in which the NotI-ApaI fragment containing a streptomycin resistance cassette has been replaced by a ~1 Kb NotI-ApaI fragment derived from pKanWor containing a Km-resistance-cassette (aminoglycoside phosphotransferase COG3231) (Supplementary Table S1).

PCR reactions mixes contained 200 µM dNTPs, 0.5 µM of the forward and reverse primers, 0.02 U/µl Q5 High-Fidelity Master Mix (New England Biolabs) and template DNA: 100 ng/µl *Xenorhabdus* genomic DNA (isolated with Invitrogen PureLink™ Genomic DNA Mini Kit) or 20-50 ng/µl plasmid DNA (purified with Wizard® plus SV minipreps DNA Purification systems). The thermocycling conditions were an initial denaturation at 98°C for 3 min, 35 cycles of 98°C for 10 s, annealing for 30 s, extension at 72°C (1 Kb/30 s), followed by a final extension at 72°C for 2 min. The PCR products were then analysed by agarose gel electrophoresis. To enrich linear, amplified pKNGkan and prevent carryover transformation of the circular template, PCR was first performed on serial dilutions of 100 ng/µl pKNGkan.

The lowest concentration yielding positive PCR results was then subjected to overnight DpnI digestion (8 µl PCR product, 1 µl DpnI [NEB R0176S/L] and 1 µl nuclease-free water) at 37°C, which should cleave cell-derived, methylated, circular, but not amplified, unmethylated, linear DNA. The DpnI enzyme was then inactivated at 80°C for 20 min. All linearized PCR products, including DpnI-digested pKNGKan, were purified, concentrated, and purified using a Zymo DNA Clean & Concentrator Kit. For each deletion construct, upstream genomic fragments, downstream genomic fragments, and linearized pKNGKan were combined in equimolar concentrations with HiFi master mix and incubated at 50°C for 1 h. The resulting mix was transformed into *E. coli* S-17 λ pir dap-dependent cells using the NEB High Efficiency Transformation Protocol (C2987H) V1 and positive transformants were selected for on LB with DAP and kanamycin. Briefly, 5 µL of the plasmid construct was added to 50 µL of *E. coli* S-17 λ pir DAP-dependent cells. This mixture was incubated on ice for 30 min and then heat-shocked at 42°C for 30 s. The mixture was further incubated on ice for 3 min. Next, 950 µL of recovery LB medium containing 0.02% DAP was added to the cells. The bacterial cells were cultured at 37°C with shaking for 1 h. The transformants were selected for on LB supplemented with 0.02% DAP and kanamycin (50 µg/mL final concentration), since the *E. coli* cells used for this transformation depend on DAP for growth, and the pKNGkan plasmid contains a kanamycin resistance gene. Transformants were assessed by colony PCR using insert-specific primers (forward primer of the upstream sequence and reverse primer of the downstream sequence) (Supplementary Table S2). Plasmids were extracted from the positive clones and further verified by DNA sequencing (Plasmidsaurus).

*X. nematophila* were transformed with plasmid constructs by conjugation with *E. coli* donor cells. Briefly, 5 mL LB supplemented with 0.02 % DAP and kanamycin (50 µg/mL final concentration) was inoculated with a single colony of the donor *E. coli* and while LB only was inoculated with recipient *X. nematophila* ATCC19061 (HGB800). Cultures were incubated overnight at 37 °C and 30 °C, respectively, with shaking until late-log phase growth ($OD_{600}$ = 0.8). Cells were harvested by centrifugation at 13,000 x ***g*** for 10 min at room temperature, washed twice with phosphate buffered saline (PBS), and resuspended in 500 µL PBS. The donor and the recipient cells were mixed in equal ratios and spotted onto LB plates supplemented with DAP to support the growth of S17-1 λ*pir* DAP-dependent *E. coli* and facilitate transfer of the deletion construct.

The conjugation mixture was incubated overnight at 30 °C, resuspended in LB, and plated onto LB agar supplemented with kanamycin (50 µg/mL final concentration). The absence of DAP eliminated the donor strain, while kanamycin selected for exconjugants (merodiploids) with the deletion vector. Post-conjugation, exconjugants in which the plasmid had integrated into the chromosome were selected for on LB agar with kanamycin. Colonies were confirmed for vector integration via PCR using insert-specific primers (Supplementary Table S2). Exconjugants were streaked on LB with kanamycin for purification, then on sucrose-containing LB without NaCl to promote excision of the *sacB*-kanamycin suicide vector and the wild type allele, yielding deletion mutants. Kanamycin-sensitive, sucrose-resistant colonies were PCR-verified for deletion events using flanking confirmation primers (Supplementary Table S2) and further verified by sequencing.

***xnpH1* complementation**

To complement the tail fiber gene (*xnpH1*) of ATCC19061 in the *X. nematophila* ATCC19061-Δ*xnpH1* and Anatoliense-Δ*xnpH1* mutants, pEVS107 donor plasmid was modified to deliver *placUV5*-*xnpH1* (*xnpH1* under the constitutive *placUV5* promoter) into the chromosomal *att*Tn7 site of the two mutants. First, a modified plasmid (pURR25::*placUV5_xnpH1*) was generated by replacing the GFP reporter, which in pURR25 is expressed from the constitutive placUV5 promoter, with *xnpH1* (XNC1_1193). Briefly, the pURR25 backbone lacking GFP was PCR-amplified with primers EA199

and EA200 using Q5 DNA polymerase (NEB), digested with DpnI, and purified (Zymoclean Gel DNA Recovery Kit, Zymo). The *xnpH1* DNA fragment was amplified from *X. nematophila* ATCC19061 genomic DNA with primers EA201 and EA202 and purified (DNA Clean and Concentrator Kit, Zymo). The backbone and *xnpH1* fragments were assembled using HiFi Assembly Master Mix (NEB); this assembly placed *xnpH1* immediately downstream of placUV5 in the resulting plasmid. The assembled plasmid was used as the template to PCR-amplify the *placUV5–xnpH1* cassette with primers EA183 (placUV5 forward) and EA180 (xnpH1 reverse2), and the amplified cassette was confirmed by agarose gel electrophoresis and sequencing. For cloning into the Tn7 delivery vector, pEVS107 was PCR-linearized (with primers EA203 and EA204), digested with DpnI, purified (Zymoclean Gel DNA Recovery Kit, Zymo), and assembled with the *placUV5–xnpH1* cassette using HiFi Assembly Master Mix (NEB). The assembled construct was transformed into *Escherichia coli* S17 λpir dap-dependent cells, and transformants were selected on LB agar supplemented with DAP and kanamycin.

Recombinant clones were screened by colony PCR using *placUV5–xnpH1*-specific primers, EA183 (placUV5 forward) and EA202 (*xnpH1* reverse), and the insert was verified by sequencing.

Conjugation of the pEVS107::*placUV5–xnpH1* construct into *X. nematophila* ATCC19061-*ΔxnpH1* and Anatoliense-Δ*xnpH1* was performed following an established Tn7 delivery protocol [2]. Briefly, overnight cultures of the *E. coli* donor strain carrying pEVS107::placUV5–xnpH1, the *E. coli* helper strain carrying the Tn7 transposase plasmid (pUX-BF13), and *X. nematophila* ATCC19061-*ΔxnpH1* and Anatoliense-Δ*xnpH1* recipient strains were grown in LB supplemented with the appropriate antibiotics and 0.02% DAP for the DAP-dependent *E. coli* strains. LB media were inoculated with each strain (10%) and grown to an OD600 of ~1.0. Cells were then mixed at a donor:helper:recipient ratio of 1:1:3 (300 µL donor, 300 µL helper, 900 µL recipient), pelleted, and resuspended in 250 µL LB. 30 µL from the mixture was spot-plated onto LB agar supplemented with 0.1% pyruvate (LBP) and 0.02% DAP and incubated overnight at room temperature in the dark. Following conjugation, bacteria were streaked onto LBP plates containing 200 µg/mL (final concentration) erythromycin to select for transconjugants. Candidate colonies were further purified on LBP erythromycin, and single colonies were grown in LB + erythromycin for glycerol stock preparation and genomic DNA extraction (Purelink Genomic DNA Mini Kit, Invitrogen). Correct integration of the ***placUV5–xnpH1* cassette** at the chromosomal *att*Tn7 site was confirmed by PCR with *att*Tn7-insertion confirmation primers (EA225; universal_glmS_Fwd_1 and EA226: Xnem_Tn7downstream_Rev) followed by sequencing.

**Table S1. Strains and plasmids used in this study**

| ***Xenorhabdus nematophila* strains** | | | | | | |
|---|---|---|---|---|---|---|
| **Strain** | **Genotype** | **HGB number** | **Nematode host** | **Geographic origin** | **Genome Accession/Locus tag** | **Source/Reference** |
| ATCC19061 | Wild type | HGB800 | *S. carpocapsae* Weiser DD-136 | USA | GCF_000252955.1 | ATCC |
| | Δ*xnpH1* | HGB2724 | | | XNC1_1193 | This study |
| | Δ*xnpS1* | HGB2766 | | | XNC1_1206 | This study |
| | Δ*xnpH1* Δ*xnpS1* | HGB2767 | | | | This study |
| | *ΔxnpH1:attTn7: placUV5–xnpH1* | HGB2774 | | | | This study |
| Anatoliense | Wild type | HGB1418 | *S. anatoliense* | Turkey | GCF_000820905.1 | P. Stock; Hazir *et al.,* 2003 |

| | | | | | | |
|---|---|---|---|---|---|---|
| | *ΔxnpH1* | HGB2762 | | | XNA1_v220011 | This study |
| | *ΔxnpH1:attTn7: placUV5–xnpH1* | HGB2775 | | | | This study |
| AN6/1 rif[R] | Wild type | HGB081 | *S. carpocapsae* | USA | GCF_000953355.1 | S. Forst |
| F1 | Wild type | HGB1849 | *S. carpocapsae* Plougastel | France | GCF_000389595.1 | P. Tailliez; Lanois *et al.*, 2013 |
| C2-3 | Wild type | HGB2299 | *S. carpocapsae* | Republic of Korea | GCF_000785665.1 | S.-J. Hun and J.-H. Shin; Hong *et al.*, 2015 |
| Websteri | Wild type | HGB1419 | *S. websteri* | Peru | GCF_000820925.2 | S.P. Stock; Nguyen 2007 |

| **Other bacterial strains** | | |
|---|---|---|
| **Lab Strain Number** | **Description/Use** | **Source/Reference** |
| HGB1261 | BW29427 *Escherichia coli* S-17 λ pir dap-dependent strain | K.A. Datsenko and B.L. Wanner |
| HGB2432 | *E. coli* BW29427 (HGB1261) containing helper pUX-BF13 plasmid for Tn7 transposition | H. Goodrich Blair |
| HGB280 | *E. coli* CC118 lamda pir containing Tn7 delivery vector, pEVS107 | H. Goodrich-Blair |
| HGB1262 | *E. coli* BW29427 Tn7 GFP containing pURR25 plasmid which was used as the source of placUV5 promoter | H. Goodrich Blair |
| HGB0801 | *Photorhabdus luminescens* TT01 | András Fodor |
| **Plasmids** | | |
| **Name** | **Description/Use** | **Source/Reference** |
| pKanWor | pBluescript II KS+ (Stratagene) with Km cassette (1 Kb) in BamHI site. HGB0181 | E. Vivas and H. Goodrich-Blair |
| pKNGKan | pKNG101 derivative with the Str cassette replaced by a Km cassette from pKanWOR. HGB1373 | E. Herbert and H. Goodrich-Blair |
| pKNGKan -ΔXNC1_xnpH1 | pKNGKan carrying *X. nematophila* ATCC19061 Δ*xnpH1* gene construct flanking the multiple cloning site. HGB2723 | This study |
| pKNGKan -ΔXNC1_xnpS1 | pKNGKan carrying *X. nematophila* ATCC19061 Δ*xnpS1* gene construct flanking the multiple cloning site. HGB2765 | This study |
| pURR25 | Source of the constitutive placUV5 promoter. HGB1262 | H. Goodrich-Blair |
| pURR25::*placUV5_xnpH1* | pURR25 carrying *X. nematophila* ATCC19061 Δ*xnpH1* gene under placUV5 promoter | This study |
| pEVS107 | Tn7 delivery vector. HGB280 | H. Goodrich-Blair |
| pEVS107::*placUV5–xnpH1* | pEVS107 carrying *X. nematophila* ATCC19061 Δ*xnpH1* gene under placUV5 promoter. HGB2773 | This study |
| pKNGKKan -ΔXNA1_xnpH1 | pKNGKan carrying *X. nematophila* Anatoliense Δ*xnpH1* gene construct flanking the multiple cloning site. HGB2761 | This study |

## Table S2. Primers used in this study

| Primer Name | Sequence (5'-3')[a,b] | Use |
|---|---|---|
| XNC1_ΔxnpH1 UPFOR (EA27) | ctgcgtgcaatccatcttgCCAAAGAACTGTTGGAAATC | Amplify upstream fragment for XNC1_1193 deletion |
| XNC1_ΔxnpH1 UPREV (EA28) | cgaaagccaagagtataccGGTCTAATCCTCATGTCATC | Amplify upstream fragment for XNC1_1193 deletion |
| XNC1_ΔxnpH1 DOWNFOR (EA29) | GGTATACTCTTGGCTTTCG | Amplify downstream fragment for XNC1_1193 deletion |
| XNC1_ΔxnpH1 DOWNREV (EA30) | ggatcgtttcgcatgattgaaGTTGTAACTACCAAAGATGGC | Amplify downstream fragment for XNC1_1193 deletion |
| XNC1_ΔxnpH1 ConfirmFOR(EA51) | CTGTTAGAAGAGCGTAAAAGAG | Confirm XNC1_1193 deletion |

| | | |
|---|---|---|
| XNC1_∆xnpH1 ConfirmREV(EA52) | GTTTAGTCCGTATCCATATGC | Confirm XNC1_1193 deletion |
| XNC1_∆xnpS1 UPFOR (EA104) | ctgcgtgcaatccatcttgGTTTGTCCTGTGGCCATG | Amplify upstream fragment for XNC1_1206 deletion |
| XNC1_∆xnpS1 UPREV (EA105) | gtaatgccatcagttagccccATTAGCGGTTCTCCTGTTAAGG | Amplify upstream fragment for XNC1_1206 deletion |
| XNC1_∆xnpS1 DOWNFOR (EA106) | GGGGCTAACTGATGGCATTAC | Amplify downstream fragment for XNC1_1206 deletion |
| XNC1_∆xnpS1 DOWNREV (EA107) | ggatcgtttcgcatgattgaaCAGGACAAGCAGCATAGAATCC | Amplify downstream fragment for XNC1_1206 deletion |
| XNC1_∆xnpS1 CONF_FOR(EA166) | GTCCTGCATCTAGACCAC | Confirm XNC1_1206 deletion |
| XNC1_∆xnpS1 CONF_R (EA167) | CTCCCCCTAGTCACATAG | Confirm XNC1_1206 deletion |
| XNA1_∆xnpH1 UPFOR (AT1) | ctgcgtgcaatccatcttgGCGTGTCGAGAAATGATGTC | Amplify upstream fragment for XNA1_v220011 deletion |
| XNA1_∆xnpH1 UPREV (AT2) | aatggttaccaaattaggaaaattcacag | Amplify upstream fragment for XNA1_v220011 deletion |
| XNA1_∆xnpH1 DOWNFOR (AT3) | ctgtgaattttcctaatttggtaaccattGGTCTAATCCTCATGTCATC | Amplify downstream fragment for XNA1_v220011 deletion |
| XNA1_∆xnpH1 DOWNREV (AT4) | ggatcgtttcgcatgattgaaGAAACACTATGTCGAAGCAC | Amplify downstream fragment for XNA1_v220011 deletion |
| XNA1_∆xnpH1 CONF_FOR (AT5) | CTATCATGTTGCTTGTCCAAATAC | Confirm XNA1_v220011 deletion |
| XNA1_∆xnpH1 CONF_R (AT6) | GAGCCACTGGATTACGAAC | Confirm XNA1_v220011 deletion |
| pURR25_F (EA199) | ATGCGTAAAGGAGAAGAACTTTTCAC | Linearize pURR25 backbone |
| pURR25_R (EA200) | GCTTAATTTCTCCTCTTTAATTCTAGATGTG | Linearize pURR25 backbone |
| xnpH1_F (EA201) | ttaaagaggagaaattaagcATGAGTACCAAATTCTTTGCGC | Amplify xnpH1 |
| xnpH1_R (EA202) | agttcttctcctttacgcatTTATGTCGTCCGATACCATCC | Amplify xnpH1 |
| placUV5_F (EA183) | ggccctaggcgcgcCTCGAGCTCGAGAAAATTTATCAAAAAGAG | Amplify placUV5_xnpH1 |
| xnpH1_R2 (EA180) | cctgcagagatctactagtTTATGTCGTCCGATACCATCC | Amplify placUV5_xnpH1 |
| pEVS107_F (EA203) | ACTAGTAGATCTCTGCAGGATATC | Linearize pEVS107 |
| pEVS107_R (EA204) | CTCGAGGCGCGCCTAG | Linearize pEVS107 |
| universal_glmS_Fwd_1 (EA225) | ATCATCTCCCTGCCACACG | Confirm xnpH1 insertion at the atTn7 site |
| Xnem_Tn7downstream_Rev (EA226) | GTTAATGCCTCTTTCAGTTTGGGAGC | Confirm xnpH1 insertion at the atTn7 site |

[a]The underlined lowercase sequences in UPFOR and DOWNREV primers are overlaps homologous to the pKNGkan vector. The underlined lowercase sequences in UPREV and DOWNFOR primers are overlaps homologous to the sequences flanking the upstream and downstream regions of the genomic DNA to be deleted. These overlaps enable seamless fusion during HiFi assembly. Insert specific primers used to confirm the integration of insert-carrying vector include the UPFOR and DOWNREV for each deletion.

[b]The non-underlined lowercase sequences in xnpH1_F and xnpH1_R primers are overlaps homologous to the pURR25 vector. The non-underlined lowercase sequences in and placUV5_F and xnpH1_R2 are overlaps homologous to the pEVS107 Tn7 delivery vector. These overlaps allow for seamless fusion during HiFi assembly

## Supplementary Figures

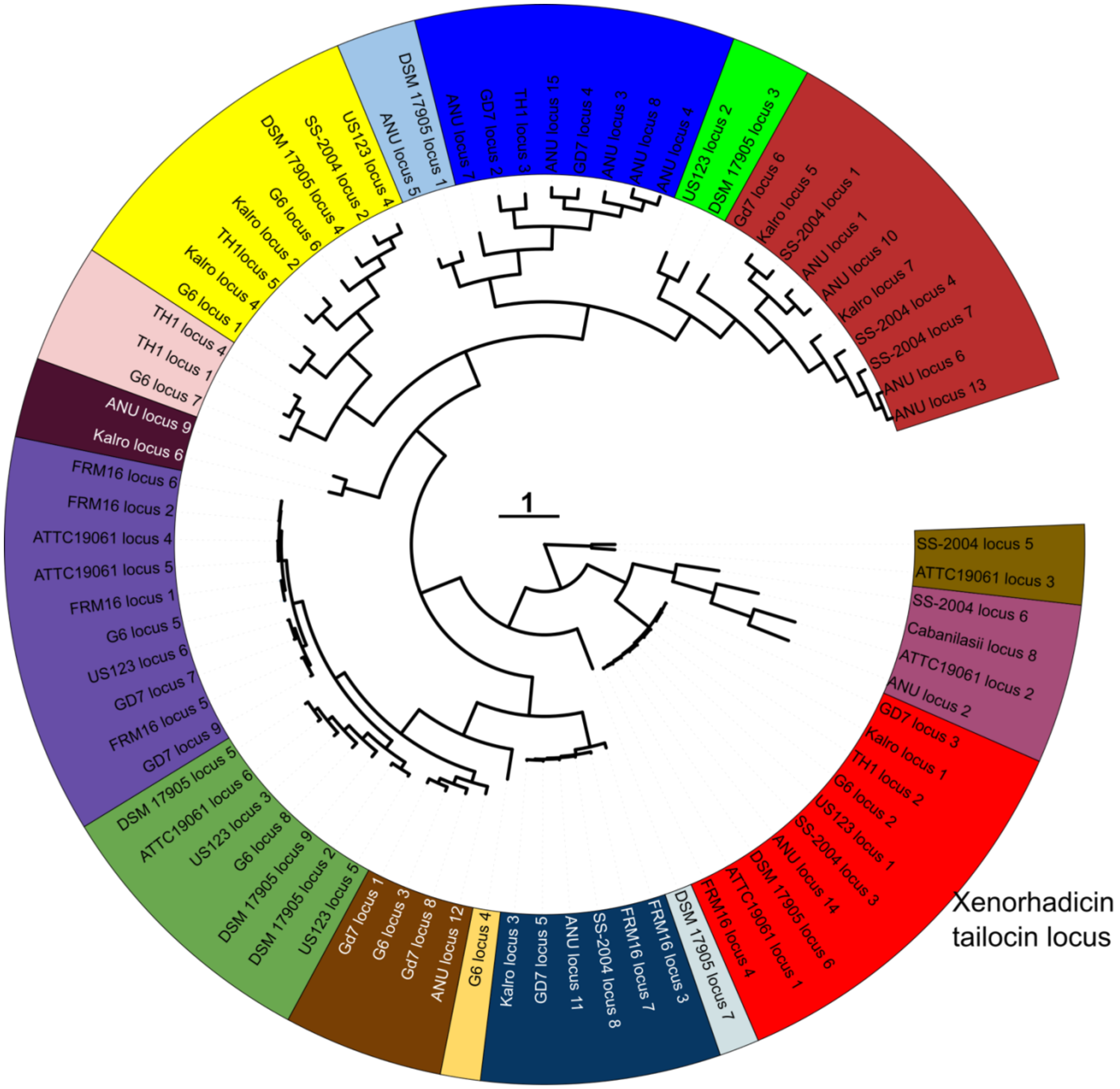


*Xenorhabdus bovienii*: SS-2004 *X. ishibashii*: GD7
*X. cabanillasii*: DSM17905 *X. nematophila*: ATCC19061
*X. doucetiae*: FRM16 *X. poinarii*: G6
*X. griffiniae*: Kalro *X. szentirmaii*: US123
*X. hominickii*: ANU *Xenorhabdus* sp. TH1

**Supplementary Figure S1:** Ten *Xenorhabdus* species contain 80 prophage/phage-like loci that are genetically diverse. This is a dendrogram based on pairwise average nucleotide identity values of prophages/phage-like loci identified in *Xenorhabdus* genomes. Each colour represents a group of prophages/phage-like loci that were genetically the most similar to each other. Among the groups, the red group was the least diverse and it contained only phage-like loci that encode xenorhabdicin tailocins. Moreover, each genome contained only one xenorhabdicin encoding locus. Scale bar represents one substitution per site.

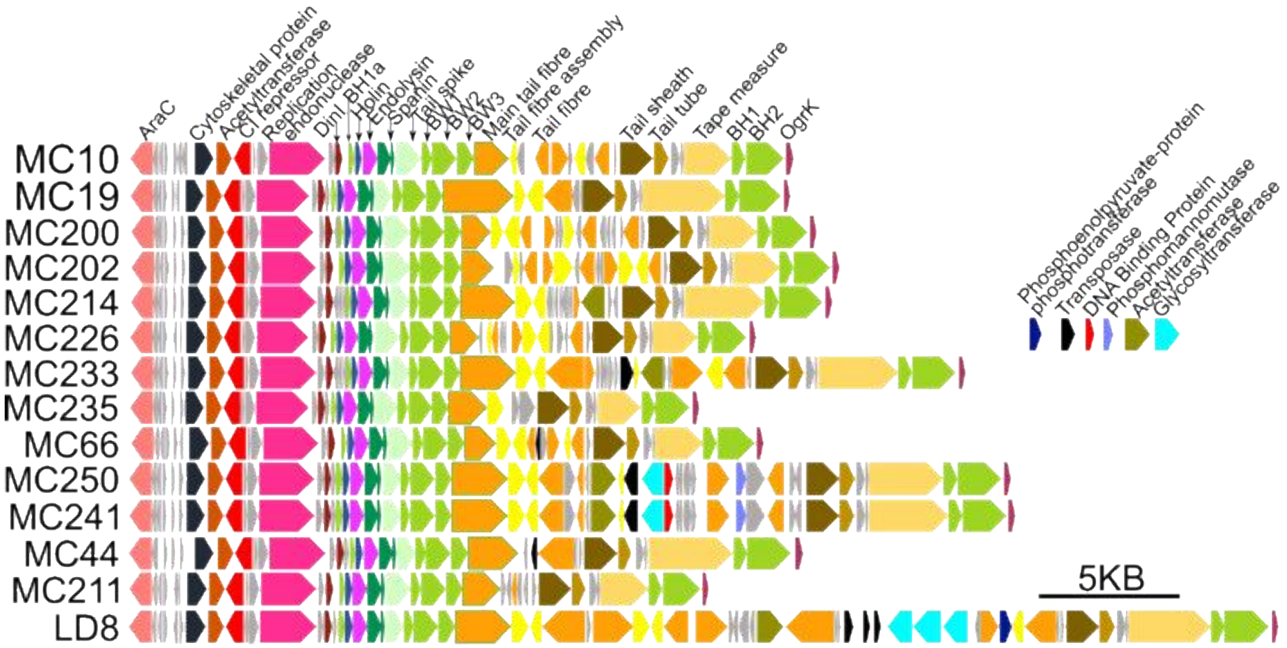


**Supplementary Figure S2: The xenorhabdicin-encoding locus (*xbp1*) in 14 strains of *Xenorhabdus bovienii* varies in length and number of tail fibre genes.** Names of *X. bovienii* strains are given in the left column. For six genes that were predicted to encode uncommon proteins, their key is given in the centre right region. Names of the predicted proteins that are encoded by the genes in the locus are shown in the topmost row. None of the loci encoded more than a singular R-type tailocin since only one tape measure-encoding gene was found per locus. Notably, the LD8 *xbp1* locus contained eight tail fibre genes and five tail fibre assembly genes, the highest number hitherto documented for an R-type tailocin locus.

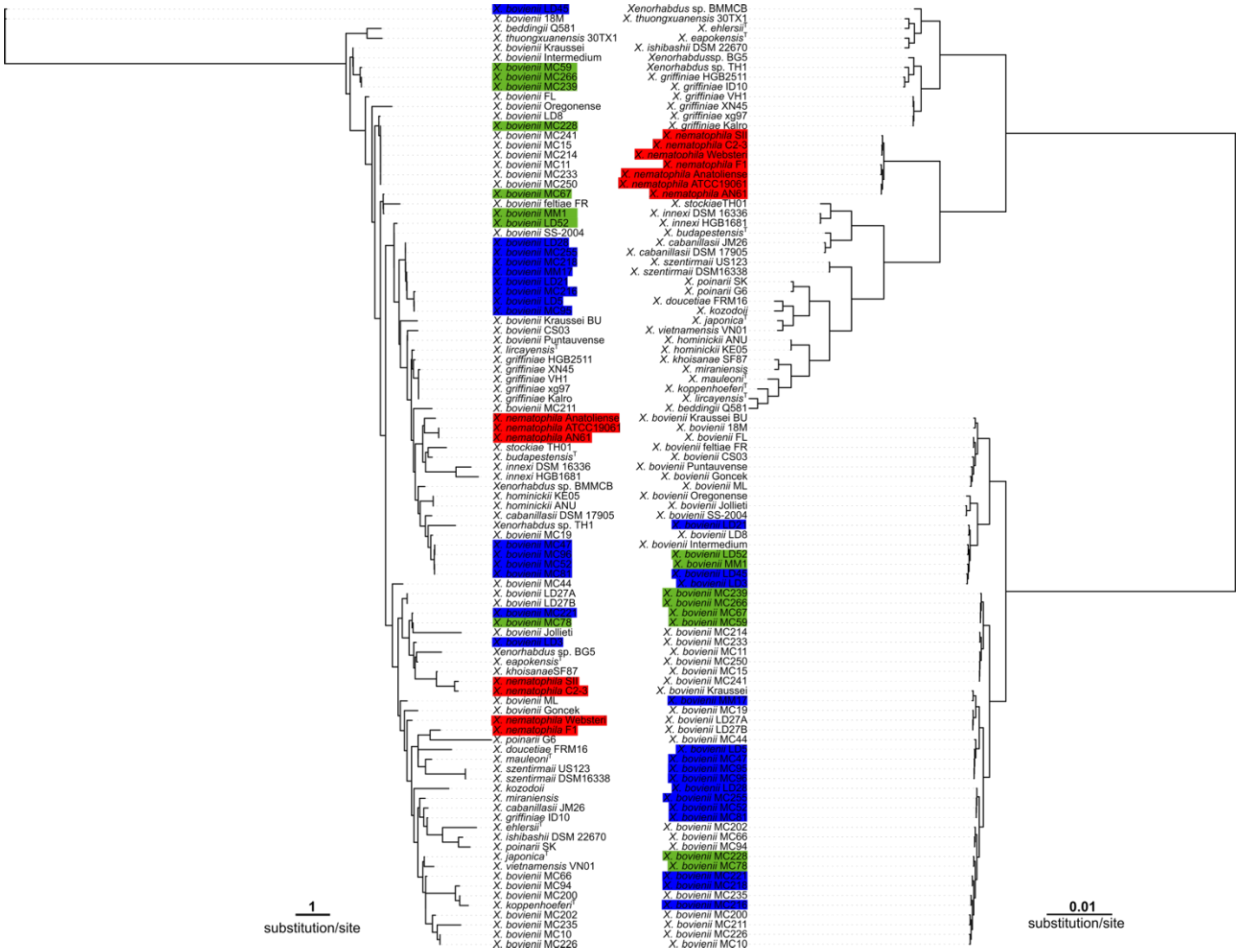


**Supplementary Figure S3:** *Xenorhabdus* xenorhabdicin tailocin main tail fibre protein sequences do not correspond to genome relatedness. On the left is a maximum likelihood tree of xenorhabdicin tailocin main tail fibre sequences while on the right is a dendrogram of genome-relatedness based on pairwise average nucleotide identities values of the same strains. Main tail fibre protein sequences did not correspond to genome relatedness since we obtained a normalized Robinson-Fould (nRF) distance of 166 out of a maximum distance of 188 when we compared an ANI-based dendrogram of the 97 strains to a maximum likelihood tree of their main tail fibre protein sequences. Strain investigated for inhibition activity are shown in red (*X. nematophila*), blue (*X. bovienii* Actor Group 2), and green (*X. bovienii* Actor Group 3).

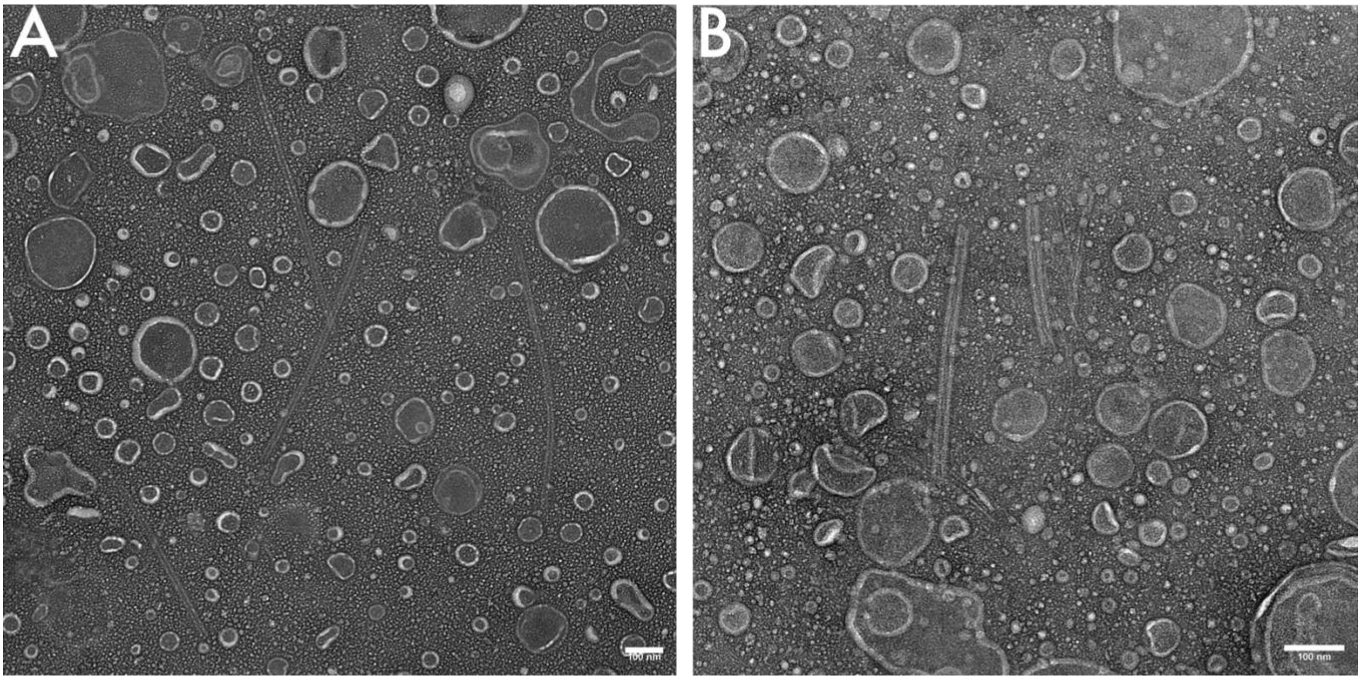


**Supplementary Figure S4:** *X. nematophila* mutants with deleted tailocin structural genes do not produce tailocins. Transmission electron micrographs of tailocin preparations from *X. nematophila* ATTC19061 whose A) main tail fibre and tail sheath genes or B) tail sheath gene only were deleted. No observable tailocin structures were observed. Scale bar represents 100 nm.

## REFERENCES


1. Kaniga K, Delor I, Cornelis GR. A wide-host-range suicide vector for improving reverse genetics in gram-negative bacteria: inactivation of the blaA gene of *Yersinia enterocolitica*. *Gene* 1991;**109**:137–141. https://doi.org/10.1016/0378-1119(91)90599-7
2. Alani OS et al. Conjugation and transposon mutagenesis of *Xenorhabdus griffiniae* HGB2511, the bacterial symbiont of the nematode *Steinernema hermaphroditum* (India). 2023. https://doi.org/10.17912/MICROPUB.BIOLOGY.000772
